# Ftl-CoV19: A Transfer Learning Approach to Detect COVID-19

**DOI:** 10.1155/2022/1953992

**Published:** 2022-07-05

**Authors:** Tarishi Singh, Praneet Saurabh, Dhananjay Bisen, Lalit Kane, Mayank Pathak, G. R. Sinha

**Affiliations:** ^1^Mody University of Science and Technology, Lachhmangarh, Rajasthan, India; ^2^Madhav Institute of Technology and Sciences, Gwalior, Madhya Pradesh, India; ^3^University of Petroleum and Energy Studies, Dehradun, Uttarakhand, India; ^4^Technocrats Institute of Technology, Bhopal, Madhya Pradesh, India; ^5^Myanmar Institute of Information Technology, Mandalay, Myanmar

## Abstract

COVID-19 is an infectious and contagious disease caused by the new coronavirus. The total number of cases is over 19 million and continues to grow. A common symptom noticed among COVID-19 patients is lung infection that results in breathlessness, and the lack of essential resources such as testing, oxygen, and ventilators enhances its severity. Chest X-ray can be used to design and develop a COVID-19 detection mechanism for a quicker diagnosis using AI and machine learning techniques. Due to this silver lining, various new COVID-19 detection techniques and prediction models have been introduced in recent times based on chest radiography images. However, due to a high level of unpredictability and the absence of essential data, standard models have showcased low efficiency and also suffer from overheads and complexities. This paper proposes a model fine tuning transfer learning-coronavirus 19 (Ftl-CoV19) for COVID-19 detection through chest X-rays, which embraces the ideas of transfer learning in pretrained VGG16 model with including combination of convolution, max pooling, and dense layer at different stages of model. Ftl-CoV19 reported promising experimental results; it observed training and validation accuracy of 98.82% and 99.27% with precision of 100%, recall of 98%, and F1 score of 99%. These results outperformed other conventional state of arts such as CNN, ResNet50, InceptionV3, and Xception.

## 1. Introduction

**Figure 1:**
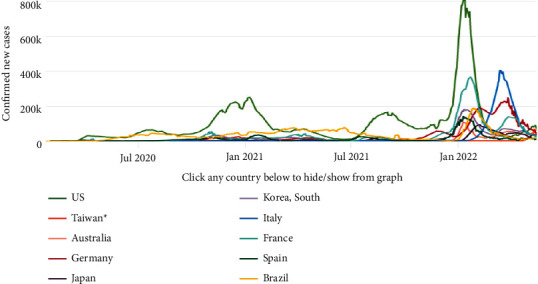
Coronavirus growth from its inception [1].

The first human trace of COVID-19 was reported in December 2019, caused through novel coronavirus. Steadily, it spreads throughout the world through various means of communication. The first human cases of COVID-19 were first reported in Wuhan City, China, in the month of December 2019. Gradually, it spreads and was declared as a pandemic by the WHO (World Health Organization) environment presented in Figure 1 [1]. Novel coronavirus disease is one such disease that infected tens of millions and affected billions of people's lives. The symptoms usually comprise of moderate fever, cough, fatigue, respiratory problems, and joint pain. Coronavirus is contagious and spreads through droplets in open environment if an infected person comes in proximity with another healthy person [2].

The coronaviruses “CoV” are a vast category of viruses that includes a series of symptoms such as the common flu to fatal respiratory illnesses. The infection ranges from symptoms such as seasonal cold, fever, cough, loss of smell, shortness in breathing, and difficulty in breathing. This risk factor of this virus increases if the patient is already subjected to serious conditions such as cancer, diabetes, obesity, and even smoking. COVID-19 not only targets the body's immune system but also the respiratory system causing acute conditions such as pneumonia, renal failure, and much more. More than million people have lost their life from the outbreak of COVID-19.

**Figure 2:**
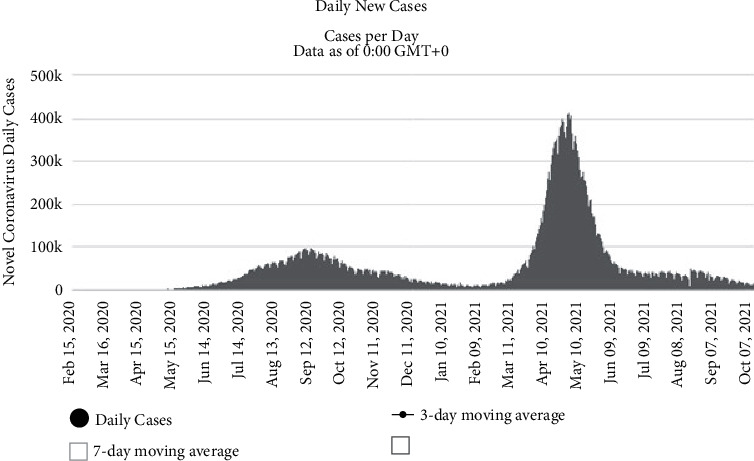
Trend of daily active COVID-19 cases as on October 20, 2021 [4].

**Figure 3:**
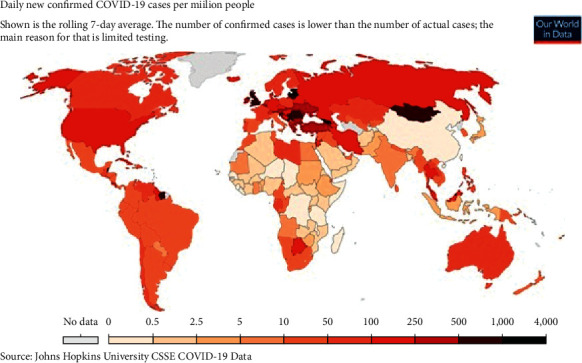
COVID-19 cases per million as on October 19, 2021 [5].

The virus has a pattern of hitting different parts of world in waves; for instance, the first wave of COVID-19 in India was recorded in September 2020, while second wave started from March 2021. Different studies pointed the various risk factors that determine the severity included age, gender, and the presence of comorbidities as important parameters [3].  Figure 2 represents the COVID-19-positive trend of daily active cases via the worldometer [4], and Figure 3 shows the daily new cases and its subsequent spread in the world [5].

### 1.1. Exposure of AI in COVID-19 Detection

Artificial intelligence (AI) applications and the importance of big data in reacting to the pandemic and preventing the long-term conditions are immense, such as detecting infected patients, monitoring the outbreak of COVID-19, advancing drug discovery, and enhancing medical care [6].

The diagnostic techniques of AI-powered COVID-19 are shown to be effective, saving radiologist time and having the correct diagnosis. For instance, one of the major complications of COVID-19 is breathlessness; it is a common symptom present in a large number of COVID-19 patients [7]. Techniques such as signal analysis, deep learning, and other classification models can be implemented to analyze the breathing pattern/sound of an individual to get an accurate feedback for the contribution to the diagnosis. Medical imaging is one of the prevailing aspects in the identification of an infection. Various models are acquired to aid in the detection of COVID-19 through chest X-ray and CT scans. Similarly, deep learning is utilized to improve the state-of-the-art functioning. CT scans detect the underlying medical conditions, which can contribute to the severity of the virus by double checking the impact on the lungs of the patient [8].

**Figure 4:**
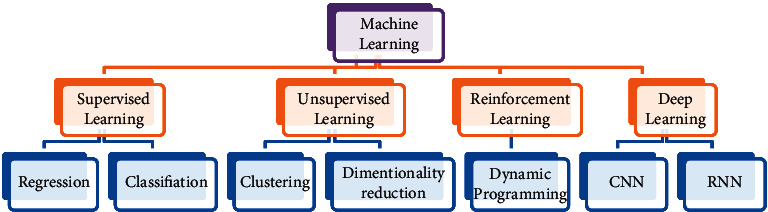
Machine learning types.

AI and machine learning algorithms have it in them, the way to find similar patterns and distinct features on a certain amount of data to then quantify them for making predictions. The sample data on which the machine trains are an important aspect in the getting accurate results. One of the primary applications of machine learning is in the healthcare system as it can be utilized to detect any disease, which is diagnosed through medical imaging such as lesions, cancer, tumors, and lung infections [9]. In the diagnostic imagining methods, chest X-ray and CT scans have played a noteworthy role in producing data regarding to COVID-19, acting as a catalyst for machine learning-based algorithms. After the COVID-19 pandemic, the trends for the medical imagining in the field of machine learning rose prominently. The models developed using various types of machine learning techniques as shown in Figure 4 are a strong alternative methods against the conventional testing mechanisms [10].

In machine learning, supervised learning is carried out on labeled data or already classified data. The division of data into training and testing makes the model more efficient as the achieved results are compared with unseen data. The degree of accuracy depends on the quality of the data, and the architecture used with the suitable classifier. Unsupervised learning utilizes unknown or unlabeled data, and thereby, it requires a vast amount of data compared with supervised learning. It classifies features and identifies patters on its own without any human touch [11]. Semisupervised learning is a common ground between supervised and unsupervised learning. It is applied when a smaller quantity of labeled data is available, which is thereby used in feature extraction from a larger unlabeled data. It provides the benefit when not having enough labeled data to train on. Cluster analysis is a technique that aims to classify a dataset into homogeneous subgroups, meaning that similar subgroups of data in each group are different from other groups. Reinforcement learning mimics artificial intelligence, and it uses the concept of behavioral machine learning along with supervised learning. Its data are not trained on the sample data, and it requires the machine to carry out hit and trial. It is performed under a complex and an unpredictable environment, where the machine learns to reward or punish based on actions performed. The ultimate target is to maximize the net rewards [12].

Therefore, that pandemic is a new outbreak, so it required high precision detection. Conventional detection techniques are not apt for its detection and involves high-end testing infrastructure. Its spread was so immense and strong that even the developed nations failed to meet the required number of test and handle the crisis. This crisis also fueled the development of new testing techniques even if they were somewhat unreliable. RT-PCR is the gold standard, but it required infrastructure and trained manpower and time to get the results. Therefore, antigen tests were conducted that gave test results very rapidly but also reported false positives and negatives [13]. COVID-19 patients' X-ray images can be used to successfully design and develop a COVID-19 detection mechanism using AI and machine learning techniques. This paper represents fine tuning transfer learning-coronavirus 19 (Ftl-CoV19), which includes the ideas of deep learning and convolutional neural networks, with visual geometry group 16 model at different stages for COVID-19 detection. Ftl-CoV19 is trained and tested on image dataset of X-rays and compared with CNN and transfer learning model for representing better image classification and accuracy. The paper is organized as: Section 2 covers the related works, Section 3 represents dataset and methods, and Section 4 presents the experimental result and analysis with comparison from current state of the art, while Section 5 concludes this paper.

## 2. Related Work

COVID-19 pandemic is a catastrophe on mankind with severe consequences on different verticals of life across the globe. The perils of this pandemic are immense and are putting down immense pressure on the social health care. Its consequences can leave a permanent scar on the fight to make the world a better and equal place. Its impact is spiral and is going to negate the already made progress in every sphere of humanity [14].

### 2.1. Conventional COVID-19 Detection Models

There are two distinct kinds of tests: diagnostic and antibody tests. A symptomatic test can show whether one has a functioning COVID-19 infection and should find a way to isolate or segregate oneself from others. There are presently two sorts of symptomatic tests—subatomic tests, for example, RT-PCR tests, for recognizing the hereditary material of the infection, and antigen tests for distinguishing explicit proteins from the infection. Antibody test comprises of conducting out tests to detect the production of antibodies against a particular infection. Apart from other symptoms, breathlessness is a common symptom noticed among COVID-19 patients.

**Figure 5:**
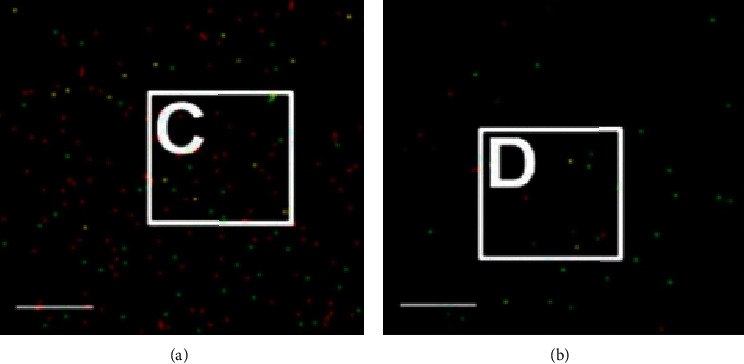
(a) Fluorescent microscopy imagery showing colocalized red and green signals as yellow particles (virus-positive). (b) Virus-negative imagery [15].

Researchers formulated the idea of virus detection using single-particle imaging and deep learning. Shiaelis et al. [15] introduced a model that detects COVID-19 and various respiratory pathogens using image analysis and convolutional neural networks, which classify the microscopic virus of respiratory illnesses based on the unique structure of each virus. The procedure begins with the extraction of virus particles through a throat swab, inactivating them, labeling of the intact virus particles by the combination of fluorescent DNAs to the surface, after which the virus particles are placed on a chitosan-coated glass slide, various distinct images are collected through microscopy imaging, and finally, the image is processed and machine learning techniques are used to classify the viruses. Viruses have distinct surface chemistry, shape, size, and structure, which enables the distribution of fluorophores on virus particle surfaces represented in Figure 5. The CNN classification segregates the virus particles based on the distribution of fluorophores on the virus surface. The CNN studies the unique characteristic distribution of fluorophores on the virus surface. The images inputted in the CNN contain virus particles illuminated as red or green fluorescence signals and yellow signals, which signify the overlap of red and green fluorescence signals. The sign of having few colocalized signals indicated COVID-19 negative, while having more colocalized was observed on virus-positive samples.

### 2.2. COVID-19 Diagnosis Using AI and Machine Learning

Artificial intelligence is studied by various research groups as a method to decipher COVID-19 and other respiratory disorders. Islam et al. [6] proposed and built a deep learning algorithm to analyze the current commercial medications, which can be repurposed in building herd immunity against COVID-19 and subsequently built applications to curb the spread of the COVID-19. Ouyang et al. [7] in another research proposed utilization of deep generative drug discovery model that is known to accelerate the identification potential drugs that can be used as the treatment. Employing the use of deep learning for computed tomography imaging, the researchers showcased the training of 499 and testing of 131 CT scans on the deep learning model alongside achieving the accuracy of 0.901. Mertz [16] introduced an AI-based technique, which have the potential to expand in detecting all sorts of respiratory illnesses and proving a versatile tool in detection. This study spotlights the various tools and technologies developed by utilizing chest X-ray and CT scans and an AI-based system called “icolung” for CT scans, which monitors the patients to sharpen the clinical view and to track the progress of the infection.

Pham et al. [17] summarized the state-of-the-art big data application that can aid in COVID-19 outbreak prediction, tracking, diagnosis, and drug discovery. Along with this, it also showcases the challenges to be overcome for the success of AI and big data-based models. Afterward, Zheng et al. [18] proposed a deep learning, software-based tool using 3D CT volumes to detect COVID-19 utilizing the pretrained UNet model for lung segmentation and taking 3D lung mask as input provides results on various thresholds and how it influences the accuracy, positive prediction rate, and the negative prediction rate.

Oh et al. [19] discussed a neural network with shallow long short-term memory (LSTM) utilized to resolve the minute dataset issues and the severity levels (extreme, low, and recovery), and the results are determined by the use of fuzzy law. Considering the recent state of the art, the most prominent architecture implementation is convolutional neural network (CNN), which is implemented in distinguishing the features in CT scans. For instance, to categorize the distinct liver masses on enhanced and augmented scans, CNN is required. While deep learning-based models have achieved a significant output for medical image abnormality diagnosis, physicians are particularly concerned about the lack of modeling, interpretability, and comprehension, which are essential for COVID-19 diagnosis. Consistent usage of CT scans puts a tremendous burden on radiology departments and possible contamination of the CT suites as the prevalence of COVID-19 increases, so the need to recognize the chest X-ray (CXR) functionality of COVID-19 is growing. As a method, CXR can be considered if the diagnostic output with CXR is enhanced to recognize COVID-19 [19]. In particular, Wang et al. [20] proposed COVID-Net, an open source deep convolutional neural network framework framed to detect the presence of COVID-19. Scans of chest radiography claimed that for COVID-19 cases with 80 percent sensitivity, COVID-Net could achieve good sensitivity. Based on the PyTorch system, the DeConVNet software was developed. The proposed system was trained in a rear-to-rear manner, implying that the CT scans were provided as the input only that the final output was supervised without any manual interference. Meanwhile, Li and He [21] illustrated deep convolutional neural network combines features and classifiers in a multilayer manner. Along with the VGG group, it comprises of the ResNet series as well. The advantage ResNet possesses over VGG models that it improves the gradient fading in when dealing with vast sets of images, thereby enabling the implementation of a deeper neural network with a better accuracy. ResNet was a leap in terms of training on deep learning networks on a massive data. ResNet50 comprises of 48 convolutional layers along with 1 max pool and 1 average pool layer often used in deep convolutional layers.

In another work, Rahimzadeh and Attar [22] presented DCNN with Xception, which introduces new inception layers, following the convolutional layers comprising of 71 layers. Wang et al. [23] in their study provided a classification based on three classes: COVID-19, pneumonia, and normal, trained on 180 X-ray images resulting a concatenated neural network of Xception and ResNet50V2 resulting in an overall average accuracy of 91.4%. This study provides InceptionV3-based deep learning model for the same using the digital image database JSRT released by the Japanese Society of Radiological Technology as medical image classification is one of the primary and widely used techniques to detect pulmonary nodules. InceptionV3 is a 48-layer deep network and requires a large dataset, but combined with transfer learning, it can be utilized on a small dataset too. In this paper, a comparative analysis between the pretrained models such as VGG17, AlexNet16, ResNet19, and NASNet with the original DCNN achieving a sensitivity of 95.41% and specificity of 80.09% on 154 images with the pulmonary nodes and 93 images without the pulmonary nodes. Summary of the state of the arts is given in Table 1.

Conventional COVID-19 techniques suffer from various constraints, and the results are not accurate that leads to panic, confusion, and its spread. Lung infection resulting in breathlessness is a common symptom among COVID-19 patients and is detected through chest X-ray. Therefore, new COVID-19 detection techniques and prediction models have been introduced based on chest X-ray images. However, due to a high level of uncertainty and lack of essential data, standard models have shown low accuracy and also suffer from overheads and complexities. In order to overcome these limitations, the next section introduces the dataset with the proposed method.

## 3. Dataset and Method (Ftl-CoV19)

**Figure 6:**
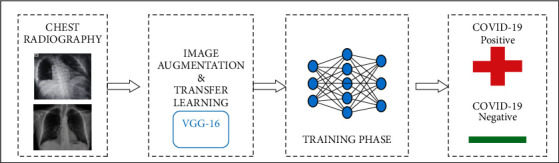
Ftl-CoV19 flow diagram.

Conventional testing methods for COVID-19 like antigen, antibody, or RT-PCR involve high-end infrastructure, costly, and time-consuming. These tests also suffer detection accuracy. Therefore, to ease the situation, many AI-based models are developed recently to predict COVID-19 presence. This section presents fine tuning transfer learning-coronavirus 19 (Ftl-CoV19) technique that will fuse the ideas of deep learning, convolutional neural networks, and visual geometry group 16. Ftl-CoV19-represented Figure 6 consists of four phases that include dataset, pre-processing, training, and detection.

### 3.1. Phase I: Dataset

**Figure 7:**
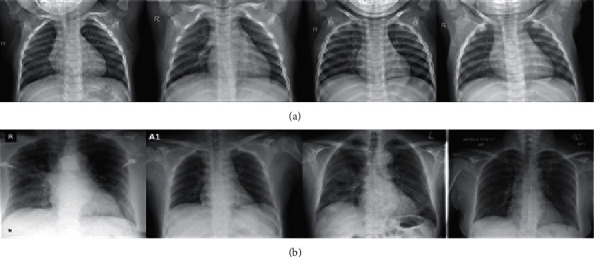
(a) COVID-19 chest X-ray; (b) normal chest X-ray [24].

Medical images in form of chest X-rays and CT scans are necessary for an automated COVID-19 diagnosis. Ftl-CoV19 in this work uses a dataset comprising of 15 publicly available datasets named “curated dataset for COVID-19 posterior-anterior chest radiography images (X-rays)” [24]. This dataset comprises of 1,281 COVID-19 and 1,481 normal posterior-anterior chest X-ray images, represented in Figure 7 [24]. The original dimensions of the images are of 450 × 456. These instances of the dataset are further augmented, trained, and tested in the next subsequent proposed phases of Ftl-CoV19.

### 3.2. Phase II: Preprocessing

Image preprocessing is widely used to balance the aspects of the image according to the needs of a model, which can improve the accuracy and prediction. It is an essential part as it affects the training time and the performance of the model, through either highlighting the essential features for the model to easily identify them, rescaling them to the appropriate size. Initially, the input images are reshaped to a desired size and then applied with various augmentation properties for training. In this phase, the images obtained from the dataset are already curated and refined from unwanted components such as noise, pixelated, compressed, medical implants, washed out image, side view CT (sliced) image, aspect ratio distortion, cropped, zoomed, rotated images, and images with annotations as represented in Table 2. The images are rotated, shifted (width and height), zoomed, and improved on rescaling, sheerness, and brightness. The details of all these operations are provided in Table 2. This preprocessing yields in the conversion of initial image of 450 × 456 into 224 × 224 to facilitate better training and validation data.

### 3.3. PHASE III: Ftl-CoV19 Training

Now in this phase, the preprocessed data are used to train Ftl-CoV19 using CNN and the pretrained VGG16, along with transfer learning and fine tuning. These techniques enable the Ftl-CoV19 to identify and classify the given chest X-ray image as COVID-19 positive or normal. Now the images are sampled and splitted as *normal_images* and *covid_images*, respectively, and then trained with batch size of 16 for training and 32 for validation, along with target size of 224 × 224 images. The whole dataset is divided in a ratio of 80 : 20 for training and testing with 2210 images for train and 552 images for validation.

#### 3.3.1. Convolutional Neural Network

**Figure 8:**
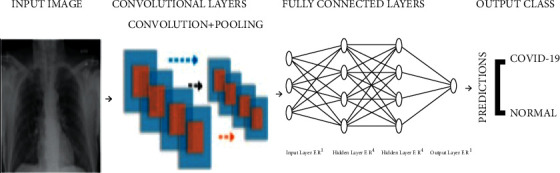
CNN architecture implementation.

Convolutional neural network (CNN) is one of the essential categories to perform image recognition and classification, and feature extraction and is commonly used in various prediction problems. The CNN image classifications take an input as an image, analyze it, and group it into certain categories as shown in Figure 8.

A CNN layer has 4 sublayers for feature extraction, convolutional layers, pooling, and various activation layers. It recognizes images in matrix form and classifies the associated pixels. CNN's forward pass algorithm is given below.

The output of the neuron of row *q* and column *r* in the “*i*”th convolutional layer with having “*q*”th feature pattern among the feature patterns as shown in the following equation:

(1) $$\begin{array}{c} O_{a,b}^{\left(i-q\right)}=\text{tanh}\left(\sum_{x=0}^{f-1}\sum_{y=0}^{q_{h}}\sum_{z=0}^{q_{w}}W_{\left(y,z\right)}^{\left(q,x\right)}O_{\left(a+y,a+z\right)}^{\left(i-1,x\right)}+{\text{Bias}}^{\left(i,q\right)}\right), \end{array}$$

where “*f*” symbolizes the number of convolutional cores.

The output of the neuron of the row “*t*” and column “*r*” in the “*i*”th subsample layer and “*q*”th feature pattern as shown in the following equation:

(2) $$\begin{array}{c} O_{t,r}^{\left(i,q\right)}=\text{tanh}\left(W^{\left(q\right)}\sum_{y=0}^{S_{h}}\sum_{z=0}^{S_{w}}O_{\left(a*S_{h}+y,r*S_{w}+z\right)}^{\left(i-1,q\right)}+{\text{Bias}}^{\left(i,q\right)}\right). \end{array}$$

The output of the “*j*”th neuron in the “*i*” hidden layer as *H* shown in the following equation:

(3) $$\begin{array}{c} O_{\left(i,j\right)}=\text{tanh}\left(\sum_{q=0}^{s-1}\sum_{t=0}^{S_{k}}\sum_{r=0}^{S_{w}}W_{\left(t,r\right)}^{\left(j,q\right)}O_{\left(t,r\right)}^{\left(i-1,q\right)}+{\text{Bias}}^{\left(i,j\right)}\right), \end{array}$$

where “*s*” is the number of feature pattern in the sample layer.

Finally, the output of the “*i*”th neuron belonging to the “*l*”th output layer as *F* is shown in the following equation:

(4) $$\begin{array}{c} O_{\left(i,j\right)}=\text{tanh}\left(\sum_{j=0}^{H}O_{\left(i-1,j\right)}W_{\left(l,j\right)}^{i}+{\text{Bias}}^{\left(i,l\right)}\right). \end{array}$$

The native convolutional layer is showcased in Algorithm 1, where the formula for the output size of convolutional filter map can be defined as shown in the following equation:

(5) $$\begin{array}{c} \text{Output size of filter map}=\left[\frac{\left(n+2\cdot p-f\right)}{s+1}\right], \end{array}$$

where *n* = size of the input image; *p* = size of the padding on input image; *f* = filter size; and *s* = stride length (^*∗*^ considering square images and filters).

The vectorized convolutional layer generalized matrix multiplication (GEMM) is presented in Algorithm 2, and because of the already present advancements and optimization in matrix dot product operations, Algorithm 2 performs better than Algorithm 1.

#### 3.3.2. Transfer Learning

**Figure 9:**
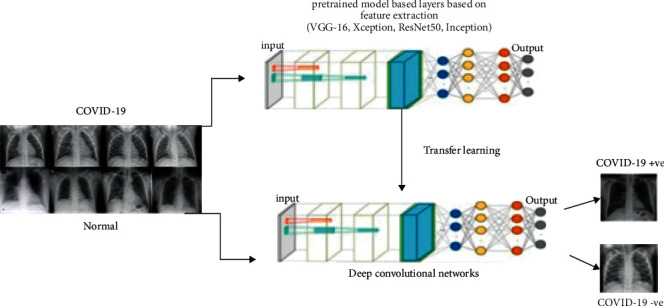
Transfer learning architecture.

Data dependency is one of the most complicated issues in deep learning, where adequate training involves a large amount of knowledge to help the network understand data patterns. In the sense of deep learning, all training and testing data are presumed to have the same distribution and feature space. In fact, appropriate training data can exist in one area, while the task of classification is carried out in another. Additionally, if the distribution of data shifts to the target domain, a complete reconstruction of the classification network with the newly collected training dataset is necessary [25]. The CNN models based on transfer learning have advantages such as limited preprocessing of the data, which are preferred, faster learning time, and time complexity can be modified by reducing the various parameters and works well on the limited dataset, making it ideal for the task of classification of medical images [25] shown in Figure 9.

In transfer learning, the source domain defined as **D**_**s**_, the learning task defined as **T**_**s**_, the goal domain written as **D**_**t,**_ and the consequent learning task defined as **Tt** along with the transfer learning have the objective to enhance the learning of the conditional probability distribution that can defined in the following equation:

(6) $$\begin{array}{c} \text{probability distribution}=P\left(\frac{Yt}{Xt}\right). \end{array}$$

The domain defined as *D* = {*X*, *P*(*X*)} has two parts:

(i) The feature space *X*
(ii) The marginal probability distributed as *P*(*X*), where *X* can be defined as the following equation:
  (7) $$\begin{array}{c} X=\left\{x_{1},x_{2},x_{3},\ldots ,x_{n}\right\}\mathrm{\in X}. \end{array}$$

If two domains are not the same, they are either following equations:

(8) $$\begin{array}{c} \left(\text{i}\right)\text{ distinct feature space}\left(X_{t}\neq X_{s}\right), \end{array}$$

(9) $$\begin{array}{c} \left(\text{ii}\right)\text{ distinct marginal distributions }\left(P\left(X_{t}\right)\neq P\left(X_{s}\right)\right). \end{array}$$

A specific given domain *D* and a task *T* = {*Y*, (.)} consist of two parts:

(i) The label space *Y*
(ii) Predictive function (*x*_*i*_, *y*_*i*_) that is not observed but is learned from training data illustrated in the following equation:
  (10) $$\begin{array}{c} \left\{\left(x_{i},y_{i}\right)i\in \left\{1,2,3,\ldots N\right\},\text{where }x_{i}\in X\text{ and }y_{i}\in Y\right\}. \end{array}$$

From a probability viewpoint, (*x*_*i*_) is written as *T* as shown in the following equation:

(11) $$\begin{array}{c} T=\left\{Y,P\left(\frac{Y}{X}\right)\right\}. \end{array}$$

Generally, if two tasks are distinct, then they may have variable label spaces (*Y*_*t*_ ≠ *Y*_*s*_) as shown in the following equation:

different conditional probability distributions defined as 

(12) $$\begin{array}{c} \left(P\frac{Y_{t}}{X_{t}}\neq P\left(\frac{Y_{s}}{X_{s}}\right)\right). \end{array}$$

#### 3.3.3. Visual Geometry Group 16

**Figure 10:**
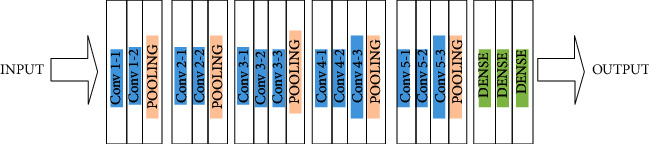
VGG architecture.

Visual geometry group 16 (VGG16) is a convolutional network for classification and detection of images. The proposed Ftl-CoV19 uses VGG-16 with 16 layers [26] represented in Figure 10. VGG16 uses a significantly huge number of parameters that concentrate on providing 3 × 3 convolution layers comprising stride 1 filter and consistently use the same padding and max pool layer of 2 × 2 stride 2 filters. This arrangement of convolution and max pool layers follows consistently across the entire design. It has 2 full layers (FC) subsequently by a SoftMax output. The 16 in VGG16 indicates 16 layers of weight. The network is an extensive and has about 138 million (approximately) parameters. This pretrained network is trained on more than a million images from the ImageNet database, which is fed with the training set images.

**Figure 11:**
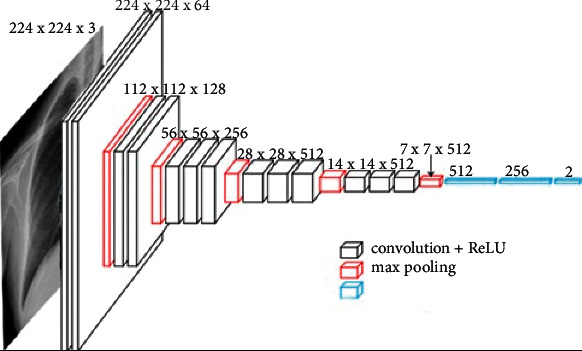
Working of the VGG model on each layer.

**Figure 12:**
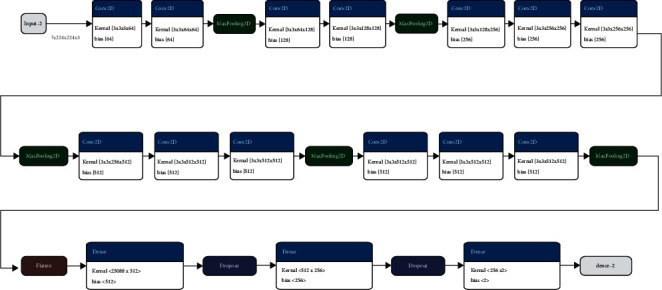
VGG architecture after fine tuning.

In Ftl-CoV19 as shown in Figure 11, the VGG16 model is used for image identification taking an input of 224 × 224 with 3-channel RGB. Before training, additional dropout and dense layers are added to the end of the pretrained VGG model. Then, the VGG16 model as shown in Figure 12 is trained on the Adam optimizer on the metrics of the accuracy of it. The Ftl-CoV19 trains on 10 epochs with the loss of 0.1198, accuracy of 96%, validation loss of 0.0755, and validation accuracy of 98%.

#### 3.3.4. Fine Tuning

Fine tuning strives to implement transfer learning by taking network as input that has already trained for a given task and then tuning or revising the network architecture to make it adjust to a specific task for a given scenario. The size of the network in the input and output layers of the existing model is modified so that it can fit the structure of the current model by adjusting the architecture, which is done by removing one or more layers from the original model and adding new layers back to do a new related job. The layers are fixed in order to keep the weights of them constant for a new model to be trained for a different task and variable data there after the new layers are fixed once the previous layers are changed [27]. In this process, only the weights of the changed layers are revised during the second training round and the weights of the constant layers are kept the same as they were after training on the original task. The modifications are made in the original model, which depends on the new dataset factors like size and the similarities to the previous data. For an instance, if two datasets have many similarities, changing the previous layer and updating with the latest one are appropriate. Therefore, transfer learning can be used as a classifier, and if in a dataset multiple layers are to be modified, it can be used as a feature extractor. Figure 12 shows layers after fine tuning in Ftl-CoV19.

The next section presents the experimental results with the proposed Ftl-CoV19, and then, comparisons are drawn with other state of arts.

## 4. Experimentation and Result Analysis

Ftl-CoV19 experimentations and comparisons with conventional CNN, ResNet50, InceptionV3, and Xception are presented in this section. Ftl-CoV19 incorporates VGG16 architecture, trained, and validated with the ratio of 80 : 20 of the dataset comprising 2210 and 552 number of instances over 5 to 25 epochs. Ftl-CoV19 is designed to detect COVID-19 to ensure the accuracy of Ftl-CoV19. The experimental results are then also evaluated through a series of metrics and the confusion matrix. The experiments are carried out with the intent to measure the performance of proposed Ftl-CoV19 to detect the presence of COVID-19 from the given set of chest X-ray images as inputs, which then extracts low-level image features such as lines, edges, and blobs and classifies them. Afterward, a comparison of the other available techniques such as CNN, ResNet50, InceptionV3, and Xception is performed with the Ftl-CoV19 under similar test conditions, which are implemented by stacking a set of convolutional layers and dropout layers to prevent overfitting. To conduct an unbiased comparative analysis between all the models, the same image augmentation properties have been applied as shown in Table 2. Afterward, confusion matrix is discussed, which embodies the evaluation of the efficiency using performance determining parameters such as accuracy, precision, recall, and specificity.

### 4.1. Experimentations

**Figure 13:**
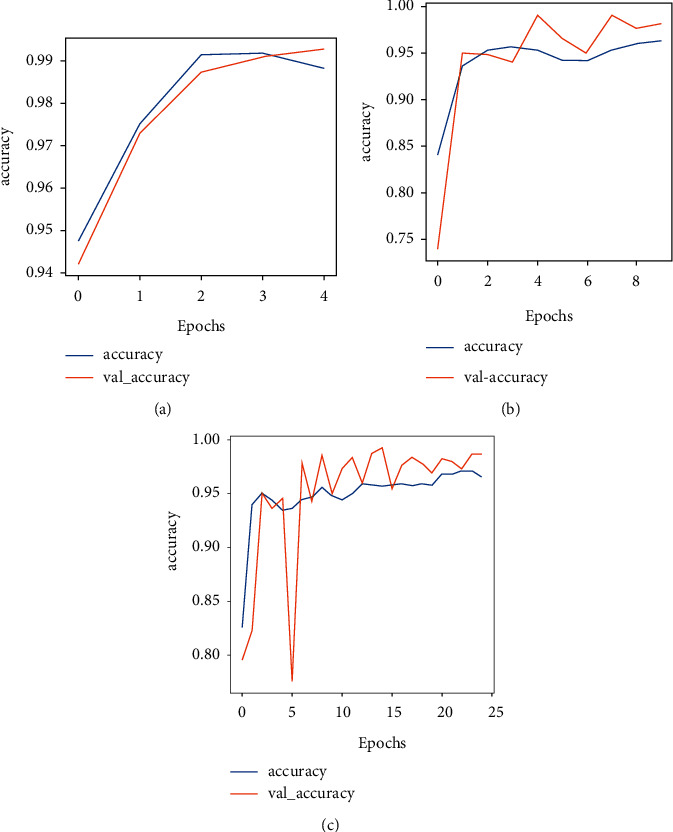
(a–c) Training accuracy and validation accuracy over 5, 10, and 25 epochs.

**Figure 14:**
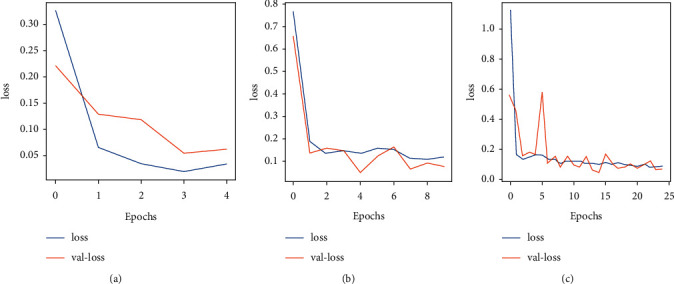
(a–c) Training loss and validation loss over 5, 10, and 25 epochs.

Ftl-CoV19 and other pretrained models (ResNet50, InceptionV3, and Xception) are trained with a dataset of 2762 images of normal and COVID-19 instances. These experiments are performed on the Google colaboratory platform. Experimental results presented in Figures 13(a)–13(c) and 14(a)–14(c) indicated the performance of (a)–(c) Ftl-CoV19 under varying epochs from 5 to 25. Figures 13(a)–13(c) reported the Ftl-CoV19 training and validation accuracy with respect to increasing epochs from 5 to 25. It reveals as the number of epochs increases the training and validation accuracy rises, which is evident from the theoretical knowledge base. Figures 14(a)–14(c) presented the training and validation loss as the number of epochs increases. This highlights the fact that the epochs increase the training and the validation loss deceases, which is in-line with the basic premise.

**Figure 15:**
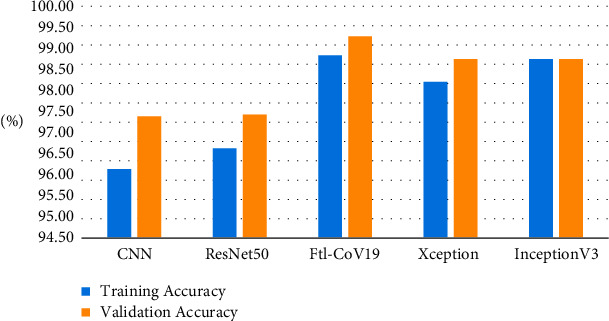
Model-wise comparison of Ftl-CoV19.

**Figure 16:**
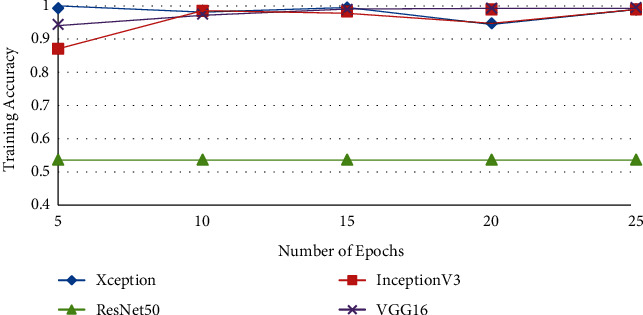
Training vs number of epochs.

**Figure 17:**
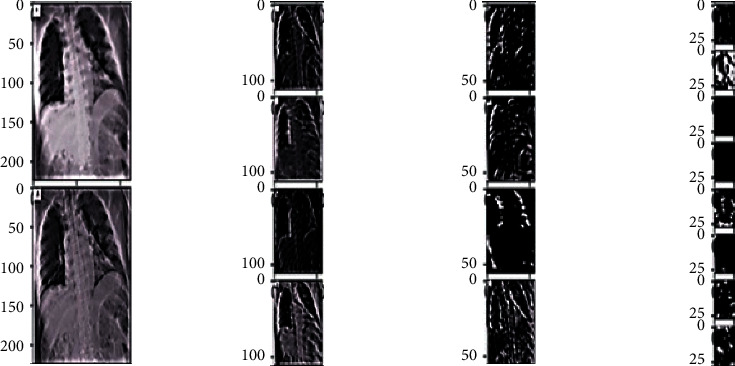
Input chest X-ray through the VGG layers (0, 3, 6, and 9).

**Figure 18:**
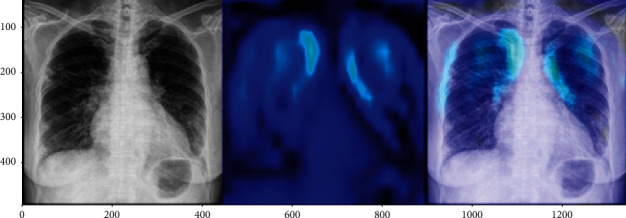
The heatmap of a COVID-19-positive chest X-ray.

In the next experiments, a comparison is between Ftl-CoV19 and other state of arts such as CNN, ResNet50, InceptionV3, and Xception, which are performed, and training and validation accuracy is obtained. In these experiments, training set and validation set sizes were 80% and 20%, respectively, of the complete dataset comprising of normal and COVID-19 instances. The experimental observation illustrated in Table 3 and Figures 15 and 16 revealed that the proposed Ftl-CoV19 reported highest training accuracy of 98.82%, while ResNet50 attained lowest training accuracy of 93.61%. Also, Ftl-CoV19 claimed the highest validation accuracy of 99.27% and ResNet50 achieving a lowest of 97.61%. These results highlighted the fact that the training and validation of Ftl-CoV19 is a success and the new integrations are enabling it to achieve high training and validation results. Experiments performed with Ftl-CoV19 and then tested against with the unseen test dataset to determine its efficiency working of it through each layer (0, 3, 6, and 9) represented in Figure 17. Lastly, Figure 18 showcases the heatmap of a sample chest X-ray through the Ftl-CoV19. It showcases the infected part highlighted in through the middle image and simultaneously overlaying it on the original X-ray in order to get a clear diagnosis.

### 4.2. Confusion Matrix

**Figure 19:**
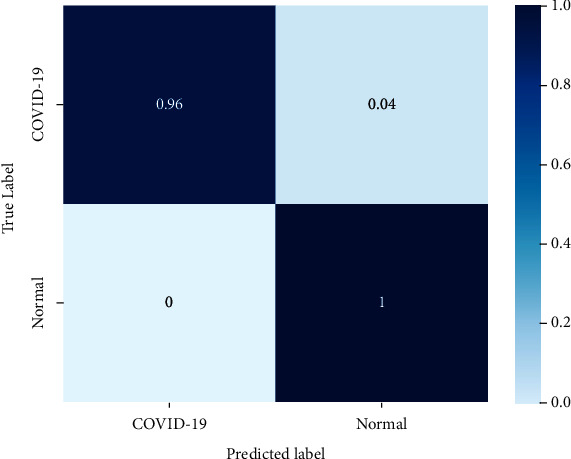
Confusion matrix for prediction of COVID-19 and normal chest scan images.

Table 4 clearly showcases the performance results in terms of the specified metrics in context to being detected as COVID-19 and normal. In Figure 19, it can be observed that *normal* category of chest images are being correctly classified with the accuracy of 100%, whereas COVID-19 is correctly classified with 96%, which is due to the smaller quantity of training samples for the class. Overall, it can be stated that it is decent accuracy for classification. From the confusion matrix, classification performance report was calculated. Four outcomes can be observed after the prediction process: true positive **(TP)**, the number of people that are accurately diagnosed as COVID-19 positive; true negative **(TN)**, the number of people that are accurately diagnosed as COVID-19 negative; false positive **(FP)**, the number of people that are misdiagnosed as COVID-19 positive; and false negative **(FN)**, the number of people that are misdiagnosed as COVID-19 negative. Ideally, the TP rate and TN rate should be close to 100%, thereby proving the correct classification. Similarly, FP rate and FN rate should be as close to 0% as possible, thereby reducing the chances of wrong detection. The classification matrices can be measured, presented in [28]:

(i) Accuracy represents the number of data instances identified correctly over the total number of data instances given.
  (13) $$\begin{array}{c} \frac{\text{TP}+\text{TN}}{\text{TP}+\text{TN}+\text{FP}+\text{FN}}\text{.} \end{array}$$
(ii) Precision, as shown in equation (14), is the ratio of the positive cases found correctly to all the positive cases expected.
  (14) $$\begin{array}{c} \frac{\text{TP }}{\left(\text{TP }+\text{ FP}\right)}\text{.} \end{array}$$
(iii) Recall/sensitivity: the instances that are correctly defined positive cases to all the real positive cases—is recall as shown in the following equation:
  (15) $$\begin{array}{c} \frac{\text{TP }}{\left(\text{TP }+\text{ FN}\right)}\text{.} \end{array}$$
(iv) F1 score: the harmonic mean of accuracy and recall is the F1 metric and is a better than accuracy showcased in the following equation:
  (16) $$\begin{array}{c} \frac{2*\left(\text{precision}*\text{recall}\right)}{\left(\text{precision}+\text{recall}\right)}\text{.} \end{array}$$
(v) Support: it is the number of true label that lies in the class, shown in the following equation:
  (17) $$\begin{array}{c} \frac{\text{ TN}}{\left(\text{TN }+\text{ FP}\right)}\text{.} \end{array}$$

Here, Ftl-CoV19 embraces the concepts of deep learning, convolutional neural networks, and visual geometry group 16 at different stages for captures of lung infection and detection from chest X-rays. These concepts are fused to better train the network and identify presence of COVID-19 in lung slices obtained through lung X-ray. Also, with the experimental results it was evident that proposed Ftl-CoV19 reported outperformed the conventional pretrained models such as ResNet50, InceptionV3, and Xception. Therefore, fusions of the concepts are working fine and able to yield better results in terms of training and validation accuracy, precision, recall, and F1 score.

## 5. Conclusion

This paper introduced fine tuning transfer learning-coronavirus 19 (Ftl-CoV19) that uses the concepts of deep learning, convolutional neural networks, and visual geometry group 16 at different stages for COVID-19 detection from chest X-rays. Experimental results highlighted the fact that a number of iterations/epochs play important role in obtaining high training and testing accuracy. In the experiments as the number of epochs increased, validation accuracy of Ftl-CoV19 improved from 48.56% to 98.82%. Performance of Ftl-CoV19 is also compared to pretrained models such as ResNet50, InceptionV3, and Xception under similar test conditions, and among all these methods, Ftl-CoV19 attained highest training and validation accuracy of 98.82% and 99.27%, respectively. These observations highlight the positive impact of the concepts of new integrations. Thereafter, confusion matrix also presents encouraging results for Ftl-CoV19 in its pursuit of COVID-19 detection, and it reports a precision of 100%, recall of 98%, and F1 score of 99%, while it observed a correct classification of 98% for normal instances. In the future or in an extended work, a number of epochs and training set size can be increased depending on the hardware and computational capabilities to realize this model as a real-time system for COVID-19 diagnosis. Furthermore, RNN and LSTM networks with optimized form can be used to identification and classification of images under similar dataset.

## Figures and Tables

**Algorithm 1:**
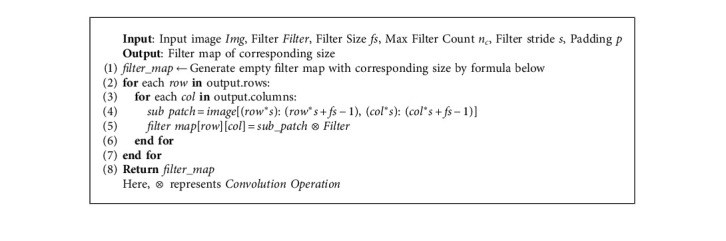
Native convolutional layer (square image and filter).

**Algorithm 2:**
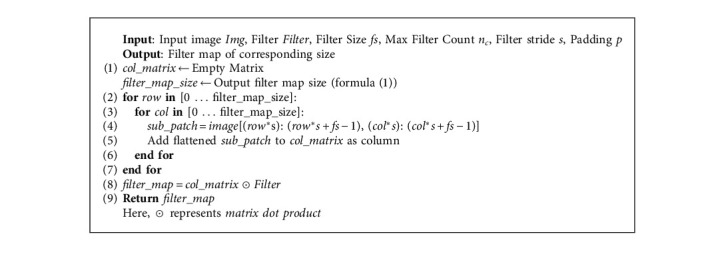
Vectorized convolutional layer generalized matrix multiplication (GEMM).

**Table 1:** Summary of the above state of the arts.

Author name	References	Highlights and contribution
Mertz	[16]	It focuses on computational-based methodologies and tools to analyze CT scans and chest X-rays like QXR
Pham et al.	[17]	Provides a compilation the state-of-the-art big data application that can aid in COVID-19 outbreak prediction, tracking, diagnosis, and drug discovery
Zheng et al.	[18]	This paper proposed a software-based tool using 3D CT volumes to detect COVID-19 utilizing the pretrained UNet model for lung segmentation
Oh et al.	[19]	An openly accessible deep convolutional neural network platform called COVID-Net with 80% sensitivity
Wang et al.	[20]	DeConVNet required training that consisted of 499 CT scans and taking over 20 hours, plotted ROC and PR curves model obtained a TPR of 0.880
Li and He	[21]	It showcases the advantage of ResNet over the VGG series due to gradient fading in identifying the shortcut connections
Rahimzadeh and Attar	[22]	It provides a classification based on three parameters such as COVID-19, pneumonia, and normal, trained on X-ray images resulting a concatenated neural network of Xception and ResNet50V2
Wang et al.	[23]	InceptionV3-based deep learning model, which results in comparative analysis between the pretrained models such as VGG17, AlexNet16, ResNet19, and NASNet

**Table 2:** Image preprocessing details.

S. no.	Operations	Details
1.	Rotation	10° clockwise and anticlockwise
2.	Width shift	0.1 fraction of the total width
3.	Height shift	0.1 fraction of the total height
4.	Zoom	0.2% smaller or larger of original image
5.	Rescale	1/255 multiplied with image channel values to the normalize input
6.	Sheerness	0.2 degrees clockwise and anticlockwise
7.	Brightness	0.25–1.0 shift value

**Table 3:** Comparative results.

	Training accuracy (%)	Validation accuracy (%)
CNN	96.12	97.37
ResNet50	96.61	97.61
**Ftl-CoV19**	**98.82**	**99.27**
Xception	98.19	98.73
InceptionV3	98.73	98.73

**Table 4:** Classification report of Ftl-CoV19.

	Precision	Recall/sensitivity	F1 score	Support
COVID-19	1.00	0.98	0.99	256
Normal	0.98	1.00	0.99	296
Accuracy	—	—	0.99	552
Macro avg	0.99	0.99	0.99	552
Weighted avg	0.99	0.99	0.99	552

## Data Availability

Data are provided in the reference.
