# Correction to “Ftl-CoV19: A Transfer Learning Approach to Detect COVID-19”

**DOI:** 10.1155/cone/9793579

**Published:** 2025-07-12

T. Singh, P. Saurabh, D. Bisen, et al., “Ftl-CoV19: A Transfer Learning Approach to Detect COVID-19”, *Computational Intelligence and Neuroscience*, vol. 2022, Article ID 1953992, 2022, https://doi.org/10.1155/2022/1953992.

In the above article, Harshul Gupta has been added to the author list, and their affiliation has been added to the affiliation list. The correct author list and affiliations are shown below.

Tarishi Singh^1^, Praneet Saurabh^1^, Dhananjay Bisen^2^, Lalit Kane^3^, Mayank Pathak^4^, G. R. Sinha^5^, and Harshul Gupta^6^

^1^Mody University of Science and Technology, Lachhmangarh, Rajasthan, India

^2^Madhav Institute of Technology and Sciences, Gwalior, Madhya Pradesh, India

^3^University of Petroleum and Energy Studies, Dehradun, Uttarakhand, India

^4^Technocrats Institute of Technology, Bhopal, Madhya Pradesh, India

^5^Myanmar Institute of Information Technology, Mandalay, Myanmar

^6^Swami Keshvanand Institute of Technology, Jaipur, Rajasthan, India

